# Effects of an Autonomy-Supportive Physical Activity Program for Compensatory Care Students During Recess Time

**DOI:** 10.3389/fpsyg.2019.03091

**Published:** 2020-01-24

**Authors:** Elisa Huéscar, Juan Antonio Moreno-Murcia, Jose F. Domenech, Juan L. Núñez

**Affiliations:** ^1^Department of Health Sciences, Universidad Miguel Hernández de Elche, Elche, Spain; ^2^Department of Sport Sciences, Universidad Miguel Hernández de Elche, Elche, Spain; ^3^Department of Psychology, Sociology and Social Work, University of Las Palmas de Gran Canaria, Las Palmas de Gran Canaria, Spain

**Keywords:** physical activity, motivation, special education students, self-determination theory, primary education

## Abstract

The purpose of this study was to examine the effects of a physical activity-based intervention conducted during recess time for Spanish students with special needs. The intervention was designed to utilize an autonomy-supportive motivational style to promote feelings of autonomy and to contribute to increased physical activity involvement in these students. Participants were 62 students in the fifth and sixth year of elementary school, with ages between 10 and 12 years (*M* = 10.75 years, *SD* = 0.80 years). Students’ perceptions of autonomy support, satisfaction of basic psychological needs, sport and physical activity motivation and actual physical activity level were assessed. A quasi-experimental design was employed with two intervention groups (autonomy-supportive and controlling styles), as well as a control group. Results indicated that students in the autonomy-supportive condition demonstrated a significant increase in feelings of autonomy and increased their physical activity levels while demonstrating a significant decrease in extrinsic motivation over the course of the intervention. The results provide support for the expectation that well-designed and theoretically based physical activity interventions can optimize learning and motivational outcomes for students in inclusive physical education settings.

## Introduction

Concern for physical inactivity among our youth has become a central issue in the educational community (Calahorro-Cañada et al., 2015). Despite increased awareness of the minimum recommendations for physical activity as provided by the World Health Organization (World Health Organization [WHO], 2010), understanding the root causes and associated problems associated with physical inactivity has attracted widespread scientific investigation (Sanz et al., 2011). In Spain, only 35% of boys and 6.3% of girls under the age of 12 years currently meet the recommended physical activity recommendations (Martínez et al., 2015), and the concern for physical inactivity constitutes a public health issue. According to the Pan American Health Organization (PHO), three-quarters of the population now lives a sedentary lifestyle that extends across all age groups (World Health Organization [WHO], 2010). One of the principal consequences of this decline in healthy lifestyles has been the increase in the development of chronic and degenerative diseases that now contribute to 68% of deaths in the world as of 2012 (World Health Organization [WHO], 2017). In contrast, physical activity involvement contributes to increased physical, psychological, social, and cognitive health benefits (Poitras et al., 2016) and physical activity promotion is thus a logical and timely recommendation to promote public health.

### Physical Activity-Based Interventions in Educational Settings

To address these concerns, various agencies have advocated for the implementation of public health interventions that are designed to promote physical activity in youth (Sigman-Grant et al., 2014). A specific objective in these types of interventions has been to reduce levels of sedentary behavior during the transition from childhood to early adolescence (Butcher et al., 2008). With respect to this purpose, various researchers have demonstrated that physical activity interventions conducted in educational settings can be effective in increasing levels of physical activity among children (Dobbins et al., 2013; Van Kann et al., 2016) while potentially contributing to favorable perceptions of competence and self-image of participants.

### Physical Activity During Recess Time

The bulk of the intervention approaches that have been conducted in the school environment have been carried out during the academic school day during physical education and recess time. The school context presents an ideal setting because this environment can be optimized in the interest of attaining curricular objectives, such as physical activity promotion. Pallasá and Méndez-Giménez (2016) and Méndez-Giménez and Pallasá-Manteca (2018) used content typical of children’s games in the design of “active recess” periods and found that it was possible to improve intrinsic motivation, enjoyment, and physical activity intentions in a sample of students in the fourth through sixth grades of elementary school. The use of sport equipment or have offered unique physical activity opportunities or games within specified play zones or designated activity areas and during recess times has been used in numerous investigations (Escalante et al., 2011; Erwin et al., 2014; Méndez-Giménez et al., 2017).

### The Social Context and Motives for Physical Activity Involvement

Despite the inherent value in utilizing recess time as a means of implementing programs that can foster students’ intrinsic motivation to engage in physical activity, as well as their enjoyment and level of physical activity involvement, sufficient research has not yet been conducted that has identified pedagogical strategies that contribute to the realization of these goals. In this regard, self-determination theory (SDT: Deci and Ryan, 2000) is a macro-theory of human motivation that is related to the functioning and development of personality within social contexts. The theory analyzes the degree to which people perform their actions at the highest level of reflection and engage in actions with a sense of choice. It represents a suitable frame of reference from which to understand individual differences in motives to engage in free-choice activities in relation to the opportunities available for the satisfaction of participants’ basic human needs and is suitable for application within educational contexts.

In relation to the conceptual framework provided by SDT, any individual’s motivation to participate in physical activity, as well as their continued adherence to physical activity, can be considered to be strongly related to their level of autonomous motivation (Friederichs et al., 2015). Researchers have examined whether teaching practices and behaviors can be modified to influence psychological need satisfaction and subsequent physical activity levels using these theoretically derived principles (Aibar et al., 2015; García-González et al., 2019). Research grounded in SDT has been particularly beneficial in addressing the importance of instructor autonomy support in contributing to student motivation for physical activity (Alcaraz et al., 2017).

An autonomy-supportive teaching style is considered to be a style of interaction that strengthens students’ personal resources and their basic psychological needs of autonomy, competence and relatedness (Reeve, 2016). This line of research is founded on a common view that autonomy-supportive teaching will strengthen intrinsic motivation and contribute to additional desired outcomes, such as psychological well-being and commitment to the activity of interest (Cheon and Reeve, 2015; Jang et al., 2016; Wang et al., 2016). Conversely, a failure to satisfy these basic psychological needs is presumed to commonly be a consequence of controlling and coercive teaching styles that is expected to result in reduced intrinsic motivation and maladaptive outcomes, such as amotivation (Haerens et al., 2016; Bartholomew et al., 2018).

### Motivation and Physical Activity Behavior in the Context of Special Education

According to federal Spanish legislation (LOE, 2006), compensatory education represents the manifestation of efforts to intervene in educational environments in such a way as to minimize disadvantages that students may encounter as a result of various underlying causes (social, economic, ethnic, etc.) that make it more difficult for certain students to receive educational access, retention and advancement. Due to their physical and psychological benefits, sport and physical activity contexts are considered desirable settings within which students can experience social and educational inclusion and related beneficial outcomes (Muñoz et al., 2017). Non-discriminatory access to all physical activity opportunities should also be respected as a right for all as expressed by various international organizations (e.g., UNESCO, 2009).

In this regard, compensatory education programs need to be designed with reference to considerations involving the social and education integration of students. There is inherent value in considering the value of pursuing these pedagogical outcomes in relation to physical education and physical activity contexts in that these settings provide opportunities for students to engage in experiential and recreational learning opportunities that may not be present elsewhere. To date, however, there has not been widespread agreement in relation to the design of intervention programs intended to yield these outcomes (Flores et al., 2015).

The purpose of the present study was to examine the effectiveness of an intervention based on SDT with the purpose of promoting physical activity during recess time for students in compensatory care. Specifically, an autonomy-supportive teaching style was contrasted with a controlling teaching style (CTS) and a control group condition with the expectation that: (1) students participating within the autonomy-supportive condition would experience greater satisfaction of their basic psychological needs of competence, autonomy and relatedness, and (2) engage in higher levels of actual physical activity in comparison to students in the CTS and control group conditions. The intervention occurred during student recess time and it is important to note that, to date, there are no known interventions that have examined autonomy-supportive interventions in the school setting for students during their recess time.

## Materials and Methods

### Sample

The sample of participants for this study consisted of 62 students (35 boys and 27 girls) between the ages of 10 and 12 years (*M* = 10.75; *SD* = 0.80) who were members of fifth and sixth grade classes and enrolled in the Spanish Compensatory Center of Special Education (CAES). The students were randomly assigned to either an autonomy-supportive condition (14 boys and 8 girls); a CTS condition (12 boys and 10 girls) or a control group condition (10 boys and 8 girls). This educational facility was composed primarily of students who were of ethnic minority and cultural backgrounds that placed them at an educational disadvantage. In addition, participants are characterized by their late incorporation into the educational system, complex educational history, family roaming or periodic educational dropouts. There are also frequent maladjustment to the school environment and the educational environment.

### Measures

#### Autonomy Support Style

To assess teachers’ need-supportive motivational characteristics, the scale of autonomy support (SAS) instrument was used as developed by Moreno-Murcia et al. (2019). This instrument is comprised of eleven items that assess a single, common factor of student perceived autonomy support in the classroom. The stem for the items on the scale is, “During activity time in class sessions of Physical Education…” (e.g., “The teacher explains to us the importance of completing these tasks”). The students respond through a Likert-type format of responses that range from “1” (“definitely not true”) to “5” (definitely true”). The pre- and post-test Cronbach’s alpha values for this scale were 0.71 and 0.70, respectively.

#### Controlling Teaching Style

The CTS as developed by Hernández et al. (2017) was used. This instrument is comprised of nine items that measure the single factor of controlling teacher style as perceived by students within physical education classes. The instrument is structured according to the stem, “In my Physical Education classes…” and students respond to items such as, “The teacher talks constantly and doesn’t permit contributions from class members” with a response range from “1” (“*definitely true”*) to “5” (“*definitely not true*”). The pre- and post-test Cronbach’s alpha values for this scale were 0.70 and 0.74, respectively.

#### Basic Psychological Needs

The Psychological Need Satisfaction in Exercise Scale (PNSE: Wilson et al., 2006) adapted to the Spanish language version (Moreno-Murcia et al., 2011) was used to assess level of psychological need satisfaction relative to competence, autonomy, and relatedness in the sport and physical activity environment. The questionnaire consists of 18 items with six items grouped across each of the three subscales: competence (e.g., “*I am confident I can do challenging exercise*”); autonomy (e.g., “*I am free to make my own exercise decisions*”); and relatedness with other (e.g., “*I get along with people that I interact with*”). Reponses to scale items are provided in relation to a seven-item Likert-type scale format ranging from “0” (“*false*”) to “6” (“*true*”) and students are asked to select the response that best conforms to their feelings about sport and physical activity. Cronbach’s alpha internal consistency values on the pre-test were 0.71 for competence, 0.82 for autonomy, and 0.63 for relatedness. On the post-test, these values were 0.81, 0.93, and 0.78, respectively.

#### Motivation

The Pictorial Scale of Sport Motivation (*Escala Pictórica de Motivación Deportiva*: Moreno-Murcia et al., in press) was utilized to assess motivation for sport and physical activity and is appropriate for youth ages 6–11 years. This instrument consists of nine items across three subscales that assess intrinsic motivation (e.g., “*I participate in sport and physical activity because I enjoy it*”); extrinsic motivation (e.g., “*To be more popular with my friends*”) and amotivation (e.g., “*I don’t like sport and physical activity*”). The response format is a three-point Likert-type scale of “1” (“*Not like me*”), “2” (“*Somewhat like me*”), and “3” (“*Like me*”). Cronbach internal consistency values on the pre- and post-test were 0.74 and 0.70 for intrinsic motivation; 0.67 and 0.66 for extrinsic motivation; and 0.74 and 0.79 for amotivation, respectively.

#### Physical Activity Involvement

The Questionnaire for Measurement of a Person’s Habitual Physical Activity (Baecke et al., 1982) in its Spanish-language version (*Cuestionario Actividad Física Habitual*: Sarria et al., 1987) was used to assess physical activity level in the participants. Leisure-time sport and physical activity involvement were assessed through four questions. The first question refers to the type of sport or physical activity or activities practiced, the frequency of practice per week, and the number of months per year during which the person engages in the activity or activities. An overall estimate was computed through the following formula: Modality 1 (Intensity × time × months practiced) + Modality 2 (intensity × time × months practiced). Specific coefficients for the various sport and physical modalities were developed according to the physical demands of the sport or physical activity in which the individual was engaged (Ainsworth et al., 2000; Florindo and Latorre, 2003). Similar questions assessed specific types of physical activity during leisure time and individuals respond according to a five-point Likert-type format. Cronbach’s alpha values on the scale for pre- and post-test were 0.71 and 0.71, respectively.

### Procedure

A quasi-experimental design was used with two intervention groups and a control group. The primary researcher received authorization (DPS.JMM.01.17) from the Center’s Director to make contact with the parents and instructors of the children and to explain the purpose of the study and to request their approval for the participation of the children. Children of those parents and instructors who provided assent were then selected for the study. The child participants were provided with a generalized description of the study’s procedures and an explanation of the nature of their involvement before being asked to provide their assent.

The autonomy-supportive and controlling teacher style groups were structured such that each group would be encouraged to recognize and value positive norms of participation in group activities. These norms included active involvement in each of the activities proposed while developing abilities to prevent and resolve conflicts; to recognize and respect individual differences; to value health and personal hygiene; and to show respect for others and for themselves in this environment. The instructional content involved group activities that required cooperation with one’s own team and the opposing team; participation in simple, choreographed individual and group dance activities; activities that had the objective of helping students to learn self-control and relaxation during the resolution of conflicts; activities that were designed to help students learn the benefits to be gained from aerobic physical activity, resistance and flexibility training; and activities that promoted learning and valuing other perspectives. Participants in the control group engaged in their customary free time activities during recess periods but did not participate in any of the activities received by the two intervention groups.

**FIGURE 1:**
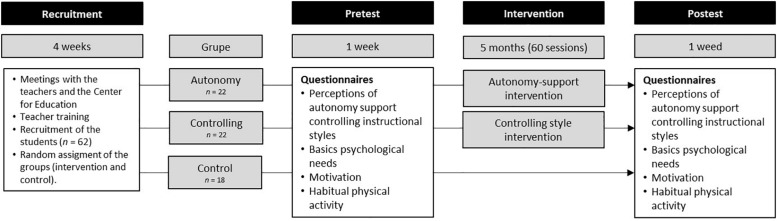
Design of the study.

The intervention was conducted during three sessions per week, each with a duration of 20 min and over the course of 5 months for a total of 60 sessions (Figure 1). Each class was structured to provide 5 min of warm up with an explanation of the activity followed by 15 min of engagement in the primary activity of the day. During the intervention, the autonomy-supportive group learned and applied a number of teaching strategies that were intended to facilitate student autonomy in the class. These strategies included a through explanation of the purpose of the activities scheduled for the day; the clarification of individual and group-level goals and challenges; an emphasis upon positive relationships among the participants and encouraging their collective involvement in the selection of tasks and decisions; encouragement of the use of language during their communication that was clear and emotionally appropriate; and encouragement of intrinsic motivation while engaged in these activities. The intention was that students would gradually develop these skills and abilities over time such that they would eventually be able to learn to participate in their own games in an autonomous fashion and to function effectively with others while involved.

A different type of intervention was implemented with the CTS group. The instructor for this group was responsible for conducting the same activities but doing so in a way in which the instructor imparted their own manner of thinking and acting rather as opposed to permitting or encouraging student engagement in the process.

The participants were all aware that they would complete questionnaires over the course of the study and they were also informed that various class sessions would be recorded on video as had been previously authorized by the school’s Board of Education. The questionnaire and video data were collected with the intention of identifying change processes that occurred over the course of the study for the three groups. When the students completed the questionnaires, they were requested to respond honestly and in an anonymous manner and they were aware that their grade in the class was in no way affected by their willingness to complete the instruments. Before the intervention was initiated, three orientation sessions of 20 min each were held for students with learning difficulties such to address any issues that may have arisen.

#### Instructional Practices

Two Physical Education teachers participated in this study. Prior to their involvement, each of the teachers participated in a workshop that described and explained the purposes of the study with regard to the adoption of the autonomy-supportive and controlling styles. During this workshop, the instructors were provided with instruction and guidance in the implementation of the corresponding styles in a way that was consistent with SDT principles (Deci and Ryan, 2002). The instructors also watched videos of teaching behaviors that corresponded with each style. Following the completion of the workshop, the teachers practiced these strategies in a pilot study in which they implemented both the autonomy-supportive and controlling teaching techniques and strategies with four classes of students of the same age and background as those that would be participating in the actual intervention. The instructors were evaluated by two observers to make sure that their teaching practices corresponded with the goals of the interventions.

Various sessions were video recorded to assess the communication and behavioral styles of these instructors in order to determine the ability to which the instructors were able to remain consistent with the instructional goals and to determine their level of correspondence with these goals. When interrater reliability levels exceeded 0.90 for the practice of each style, each teacher was considered ready to participate in the actual intervention.

An instrument developed by Sarrazin et al. (2006) that codes teaching behavior in relation to controlling, neutral and autonomy-supportive styles was used to determine the relative frequency of these types of teaching styles. During the autonomy-supportive condition, at least 80% of teacher verbalizations needed to reflect the autonomy-supportive goal whereas less than 20% of the verbalizations could be neutral or controlling in nature (Perlman, 2015).

**FIGURE 2:**
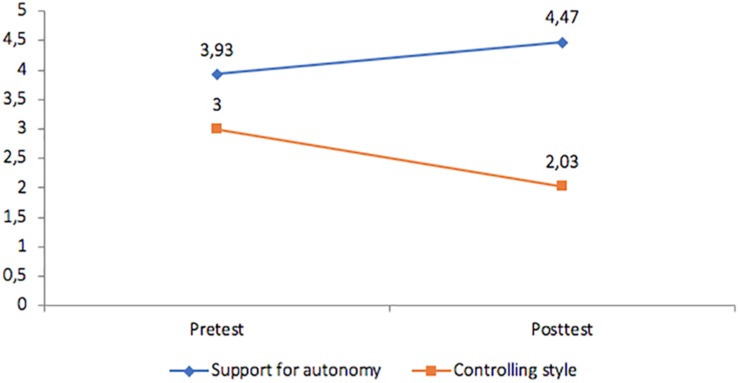
Autonomy-supportive teaching style group: changes in student perceptions of instructional style.

**FIGURE 3:**
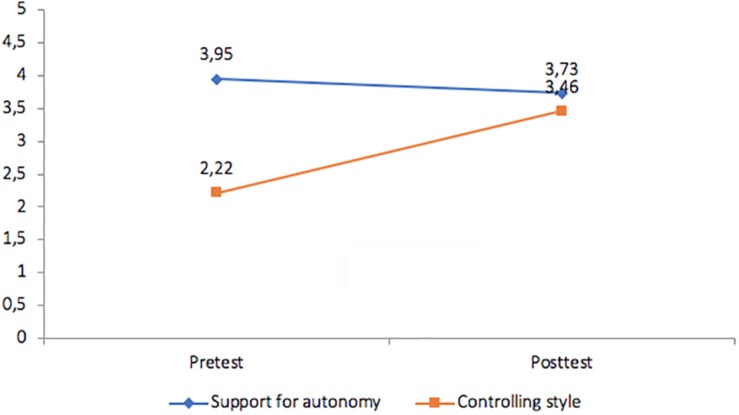
Controlling teaching style group: changes in student perceptions of instructional style.

**FIGURE 4:**
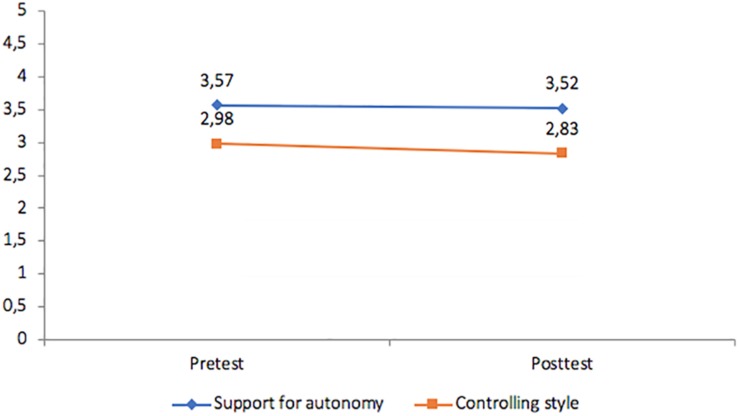
Control group: changes in student perceptions of instructional style.

To ensure that the teacher behaviors were consistent with these goals during the implementation of the intervention the students participating in the classes also were requested to provide their perceptions of the instructional style in relation to the autonomy-supportive and controlling characteristics of the verbalizations (Table 1). The purpose was to obtain an estimate of the consistency between observational data and student perceptions with regard to instructional style. A repeated measures analysis was conducted to examine the nature of the instructional styles. The results (Figures 2–4) reveal that the students in the autonomy-supported group did, in fact, perceive increasing autonomy support over time and diminished controlling behavior from their instructor and these changes were significant (*p* < 0.05). In the CTS condition, students reported stronger perceptions of teacher control and less autonomy support over time and these differences were significant (*p* > 0.05) whereas no differences in either autonomy support or instructor control were identified by students in the control condition over time.

**TABLE 1:** Instructor verbal interactions about styles over time (Times 1, 2, and 3).

Group	Comments	Time 1 (%)	Time 2 (%)	Time 3 (%)
Controlling style	Autonomy	16.6	2.2	9.1
	Control	70.8	80.6	81.8
	Neutral	12.6	17.2	9.1
Autonomy support	Autonomy	97.3	84.6	90.5
	Control	2.7	7.6	9.5
	Neutral	0	7.8	0

### Data Analysis

Descriptive data was generated that included means and standard deviations for all variables in addition to Cronbach’s alpha estimates of internal consistency for the instruments. Homogeneity of the dependent variables was examined in relation to a Levene test. A 3 × 2 repeated measures analysis of variance (RM ANOVA) was conducted on the outcome variables for the three groups at two times (pre- and post-test). In addition, teacher behavior was coded for the three classes. In addition, the behaviors of the teachers during their classes were examined using seven repeated measures in a 3 (group) × 2 (time) design with an adjusted Bonferroni *p* value of 0.01. All data were analyzed using the SPSS 25.0 program.

## Results

### Preliminary Analysis

To test for homogeneity of the three groups prior to the intervention, a single factor analysis of variance was conducted across the dependent variables (autonomy, competence, relatedness, intrinsic motivation, extrinsic motivation, amotivation, physical activity level). Wilks’ lambda statistic = 0.003, *F*(2,60) = 762.88, *p* < 0.001, η = 0.99) revealed overall group differences. Follow-up analyses revealed group differences in competence, (*F* = 7.04, *p* < 0.01, η = 0.20) between the autonomy-supported group and the control group and between the CTS group and the control group but no significant differences existed between the autonomy-supported group and the CTS group prior to the intervention. With regards to the pre-intervention levels of autonomy, values for autonomy were significantly greater for the controlling teacher style group than for the control group. Initial levels of habitual physical activity were higher in both the autonomy-supportive and controlling teacher style groups than in the control group (*F* = 4.76, *p* < 0.05, η^2^ = 0.20), but no significant differences existed between the two intervention groups.

### Intervention Outcomes

The “post-pre” changes in each of the dimensions are shown below, as well as the statistical significance of repeated measures ANOVA. The repeated measures analysis revealed that the autonomy-supportive instructional condition resulted in a significant increase in autonomy (*p* < 0.001) and in physical activity involvement (*p* < 0.01), as well as a diminishment in extrinsic motivation (*p* < 0.01) within this group. The controlling style condition resulted in decreased autonomy (*p* < 0.05) and relatedness (*p* < 0.05). To the contrary, the controlling instructional style group experienced a decrease in autonomy (*p* < 0.1) and in relatedness (*p* < 0.1) over the course of the intervention (Table 2).

**TABLE 2:** Repeated measures analysis between autonomy-supported group, controlling group, and control group.

		Autonomy-					
		support		Controlling		Control	
		(*n* = 22)		(*n* = 22)		(*n* = 18)	
		*M*	*SD*	*M*	*SD*	*M*	*SD*
Autonomy	Pre	1.81	0.92	2.87	0.65	1.43	0.51
	Post	5.18**	0.69	1.89*	0.62	1.65	0.59
Competence	Pre	5.00	0.80	5.19	0.62	4.23	0.75
	Post	4.89	0.65	4.77	0.43	4.49	0.93
Relatedness	Pre	5.60	0.38	5.43	0.45	5.17	0.80
	Post	5.78	0.94	4.87*	0.57	4.82	0.89
Intrinsic motivation	Pre	2.90	0.30	2.90	0.15	2.79	0.34
	Post	2.84	0.22	2.84	0.27	2.79	0.28
Extrinsic motivation	Pre	2.15	0.40	2.36	0.45	1.79	0.38
	Post	1.60*	0.46	1.96	0.50	1.90	0.54
Amotivation	Pre	1.18	0.34	1.15	0.34	1.44	0.51
	Post	1.21	0.30	1.18	0.34	1.40	0.43
Physical activity level	Pre	5.37	0.53	5.36	1.05	4.65	0.58
	Post	5.95*	0.79	5.18	1.75	4.84	0.81

*p < 0.05; **p < 0.01.

## Discussion

The purpose of this study was to examine the effects of need-supportive motivational style and controlling motivational style of school-aged youth in a compensatory education program during recess. Results indicated that the group that received autonomy support increased in their levels of perceived autonomy and that this support was related to greater interest in engaging in physical activity.

The need-supported treatment group demonstrated increases in self-reported autonomy over the course of the intervention. This finding suggests that the need-supportive environment facilitated student interest in physical activities that students had not previously considered for participation as their involvement was perceived as a choice rather than an obligation. No significant changes occurred with regard to the satisfaction of the basic psychological needs of competence and relatedness in the relatively short period of time in which the program was conducted. With regard to relatedness, it is possible that the shortage of students interested in involving themselves in the first choice of game selection may have impeded the optimization of the quality of the social relationships among the participants.

With regard to motivation, these results revealed a diminishment in extrinsic motivation in the need-supportive group over the course of the study which is a finding that should be kept in mind with relation to the history of learning difficulties and disruptive behaviors for these students. These students were accustomed to CTSs that focused on behavioral control and external incentives and consequences. Thus, the new approach based on autonomy support may have been the impetus by which extrinsic motivation diminished and feelings of autonomy increased over the course of the study. A related study (Lim and Wang, 2009) found that perceived student autonomy was negatively related to external regulation and amotivation. These findings are of particular relevance of teachers of students with special needs where the provision of autonomy may result in self-determined motivation and improved behavior in students. The provision of an autonomy-supportive environment over a longer period of time has proven beneficial in contributing to intrinsic motivation and enjoyment (Cecchini et al., 2012).

The intervention in this study was designed to take place with students who had limited interest in physical activity and in learning, in general. These students are also accustomed to CTSs, threats, punishment and various other forms of external regulation that can be common in such learning environments. The nature of the environment makes it difficult to achieve great change in a short period of time. Various researchers have concluded that CTSs tend to discourage students over the long term (Bartholomew et al., 2011; Aelterman et al., 2012) and can reduce any type of sustained interest in physical activity.

Additional research suggests that student choice alone is not necessarily sufficient to strengthen motivation in students in physical education classes (Sima and Einat, 2015). These findings indicate that other strategies and forms of assistance may be necessary beyond student autonomy support. Time limitations may also hinder other efforts to promote intrinsic motivation during recess time, particularly since time typically is devoted to provide explanations of the activity and to allow for each student to learn at their own pace.

Understandably, students perceive recess time as a time to relax and to disengage from cognitive demands of school which can hinder efforts during recess time. Recess time limitations can also preclude students from attaining success on tasks as well. These real world considerations can limit some of the potential gains achieved by students during recess time.

In line with the SDT that defends that self-determined motivation generates positive results (Deci and Ryan, 2000), the need-supported intervention group demonstrated an increase in physical activity level, which was an anticipated outcome in this study. This finding is also consistent with findings from related studies (Lim and Wang, 2009; Aibar et al., 2015; Wang, 2017) although these studies differed considerably in the population of interest. This outcome is also of importance because it provides support for the belief that autonomy-supportive physical activity experiences in the educational context can impact the extent to which students have an interest in physical activity involvement outside of the educational setting (Aibar et al., 2015; Moreno-Murcia et al., 2017).

Therefore, the results obtained as a whole, offer support to the SDT in the educational context in a quasi-experimental design, demonstrating in this way the importance of the social context in the positive development of the students, as reflected by the perception from the need for autonomy until the decrease in low-quality or extrinsic motivation and the increase in physical activity levels.

Certain limitations should also be noted about the present study. First, the selection of just one educational institution could be problematic and there is not a body of current knowledge on students in compensatory education with which to compare our findings. It is also recommend that future interventions be carried out over a longer period of time to determine whether the program implemented has a greater effect upon the participants. Teachers can also utilize recess time more effectively to increase levels of physical activity in children and adolescents. During this time, the physical activity sessions should be designed with considerable thought and created in such a way as to provide a high level of choice for the participants. In this way, we would anticipate that autonomy-supportive class environments will be efficacious in contributing to inclusive physical education programs. Thus, future research could help optimize the presence and relevance of this line of work through programs based on fostering social relations among students in inclusive frameworks. Teachers’ attitudes through their motivational style together with the study of existing resources or barriers through longitudinal studies could contribute to endorse in this group the knowledge we have regarding students in ordinary schools.

## Data Availability Statement

The datasets generated for this study are available on request to the corresponding author.

## Ethics Statement

The studies involving human participants were reviewed and approved by the Project Evaluation Body of the Universidad Miguel Hernandez de Elche (DPS.JMM.01.17). The patients/participants provided their written informed consent to participate in this study.

## Author Contributions

EH, JM-M, and JN: conceptualization and supervision. JM-M: methodology and visualization. EH and JD: software, investigation, and data curation. JM-M and EH: formal analysis. EH: resources. EH and JN: writing – review and editing.

## Conflict of Interest

The authors declare that the research was conducted in the absence of any commercial or financial relationships that could be construed as a potential conflict of interest.
